# Energy level as a theranostic factor for successful therapy of tissue injuries with polyphosphate: the triad metabolic energy - mechanical energy - heat

**DOI:** 10.7150/thno.100622

**Published:** 2024-08-19

**Authors:** Werner E.G. Müller, Hadrian Schepler, Meik Neufurth, Rita Dobmeyer, Renato Batel, Heinz C. Schröder, Xiaohong Wang

**Affiliations:** 1 ERC Advanced Investigator Grant Research Group at the Institute for Physiological Chemistry, University Medical Center of the Johannes Gutenberg University, Duesbergweg 6, 55128 Mainz, GERMANY; 2 Department of Dermatology, University Medical Center of the Johannes Gutenberg University, Langenbeckstraße 1, 55131 Mainz, GERMANY; 3 Galenus GH AG, Rainstrasse 7, 6052 Hergiswil, Switzerland; 4 Faculty of Natural Sciences, Juraj Dobrila University, Zagrebačka 30, 52100 Pula, Croatia

**Keywords:** Polyphosphate, Metabolic energy, Energy conversion, Thermodynamics, Wound healing

## Abstract

**Rationale:** Tissue regeneration of skin and bone is an energy-intensive, ATP-consuming process that, if impaired, can lead to the development of chronic clinical pictures. ATP levels in the extracellular space including the exudate of wounds, especially chronic wounds, are low. This deficiency can be compensated by inorganic polyphosphate (polyP) supplied *via* the blood platelets to the regenerating site.

**Methods:** The contribution of the different forms of energy derived from polyP (metabolic energy, mechanical energy and heat) to regeneration processes was dissected and studied both *in vitro* and in patients. ATP is generated metabolically during the enzymatic cleavage of the energy-rich anhydride bonds between the phosphate units of polyP, involving the two enzymes alkaline phosphatase (ALP) and adenylate kinase (ADK). Exogenous polyP was administered after incorporation into compressed collagen or hydrogel wound coverages to evaluate its regenerative activity for chronic wound healing.

**Results:** In a proof-of-concept study, fast healing of chronic wounds was achieved with the embedded polyP, supporting the crucial regeneration-promoting activity of ATP. In the presence of Ca^2+^ in the wound exudate, polyP undergoes a coacervation process leading to a conversion of fibroblasts into myofibroblasts, a crucial step supporting cell migration during regenerative tissue repair. During coacervation, a switch from an endothermic to an exothermic, heat-generating process occurs, reflecting a shift from an entropically- to an enthalpically-driven thermodynamic reaction. In addition, mechanical forces cause the appearance of turbulent flows and vortices during liquid-liquid phase separation. These mechanical forces orient the cellular and mineralic (hydroxyapatite crystallite) components, as shown using mineralizing SaOS-2 cells as a model.

**Conclusion:** Here we introduce the energetic triad: metabolic energy (ATP), thermal energy and mechanical energy as a novel theranostic biomarker, which contributes essentially to a successful application of polyP for regeneration processes.

## Introduction

The identification of theranostic factors and their application for successful treatment of patients crucially contribute to a rational causal-analytic decision chain that enables adaptive patient-specific therapy. In this study, this decision sequence is applied to the healing of chronic wounds. These wounds are characterized by their inability to heal within a period of four to twelve weeks despite established treatment 1. They develop mainly in the adult population due to venous/arterial insufficiency, neuropathies or metabolic diseases such as diabetes. The morbidity is high, the socio-economic burden is severe and the overall financial cost of wound care is substantial 2. In diabetes mellitus, the correlation between the metabolic energy supply for the organs and the state of cellular functions becomes evident. The underlying deficiency is due to an imbalance in insulin delivery. Especially type 2 diabetes caused by cellular insulin resistance and/or age-dependent impairment of insulin production results in decreased intracellular ATP synthesis and overall ATP 3, 4. Here we quantify the ATP levels in wound exudate from patients with chronic wounds in parallel to the levels in wound exudate from healthy individuals. We report that the ATP levels in chronic wounds are significantly and massively lower than in wounds from healthy subjects. In consequence, the proliferative and migratory activities of dermal fibroblasts from chronic wounds, which is physiologically high, is decreased 4, caused by an impaired Wnt/β-catenin signaling pathway, through which even an increase of energy requirements accrues 5, 6. The wound healing mechanisms, therefore, rely not only on stopping bleeding after injury but also on supply of metabolic energy to the impaired site 1, 5.

Our group asked about the physiological possibilities of supplying metabolic energy to injured sites in order to accelerate their regeneration, e.g. in wound healing. Characteristically, tissue damages are accompanied by an increase in blood supply 7. Ischemia-induced tissue damage coincides with the formation of new blood vessels 8. Consequently, the question arises, where is the extracellular ATP store or ATP generator? Even more aggravating is the fact that the extracellular ATP levels are very low at ≈10 nM, while the intracellular ATP pool is high at 3 - 10 mM (reviewed in: 9). The metabolic energy stored in each of the two energy-rich phosphoanhydride bonds of this molecule and released upon hydrolysis is 30.5 kJ/mol 10. In comparison, the metabolic energy stored in polyphosphate (polyP) with a chain length of 50 to 100 phosphate (P_i_) units is much, much higher.

Physiologically, polyP is present in an encapsulated form in the blood platelets/thrombocytes, small cell fragments that originate from megakaryocytes and are pivotal in hemostasis 11. They are filled with polyP in their dense granules, which promotes blood coagulation and inflammation and induces fibroblast chemotaxis 12-14. PolyP is an ancient molecule that has been shown to be essential for proliferation of both prokaryotic and eukaryotic cells (reviewed in: 15, 16). As a result of platelet activation, this polymer is released into the extracellular microenvironment where it becomes functionally active. PolyP is exported from the platelets either as soluble Na-polyP or as nanoparticle(NP)-associated Ca-polyP 17, 18. It has been proposed that long chain polyP polymers with a chain length of >100 P_i_ units released from activated blood platelets promote clotting of plasma via the contact pathway through factor XII 19. However, recent studies demonstrated that only short-chain polyP molecules with chain-lengths of 30 to 100 P_i_ units are released from platelets 17, which, however, elicit no significant effect on factor XII activation 20. In addition, Ca-polyP nanoparticles (Ca-polyP-NP) are released, which comprise a size of ≈500 nm (Z-average) or ≈200 nm by SEM (*scanning electron microscopy*). Physiological size Ca-polyP has been chemically prepared from Na-polyP and CaCl_2_ added in a two-fold molar excess to Na-polyP at pH 10 21.

During the enzymatic hydrolysis of the phosphoanhydride bonds, e.g. during hydrolysis of the β-γ phosphoanhydride bond within ATP, a Gibbs free energy (ΔG) of -30 kJ/mol is released 22. The enzyme alkaline phosphatase (ALP), a ubiquitous enzyme found both intracellularly and extracellularly, and associated with the outer plasma membrane surface 23, not only cleaves the P-O-P bonds in ATP 24 but also in polyP 25. As soon as it was recognized that, and this is important, in addition to heat, large amounts of metabolically usable energy are “unexpectedly” released alongside heat, as shown for muscles 26, the path to a physiological understanding of the biochemistry of energy-rich phosphate bonds was opened 27. To gain insight into the energy flow after cleavage of the phosphoanhydride bonds in eukaryotic cell systems, cell culture experiments were performed with SaOS-2 cells 28, cells capable of mineralizing to form bone-like crystals on their surfaces. Bone mineral formation, like wound healing, is an energy-requiring process 29. In both cases, the first law of thermodynamics applies, according to which energy can be converted from one form of energy to another by converting internal energy (U), the sum of kinetic (energy in motion) and potential energy (energy that can become motion), into heat (Q) and/or mechanical energy (work, W) and vice versa 30. What is important is that the total amount of energy remains constant and can neither be created nor destroyed 30. The resulting energetic relationships have been critical reviewed by A.V. Hill and O. Meyerhof using the model of muscle contraction, focusing on the relationship between mechanical function and heat production 31.

Certainly, during the enzymatic hydrolysis of the energy-rich phosphoanhydride bonds in polyP, part of the released free energy is dissipated as heat, as outlined for phosphate bonds by Lipman 22. Part of the released energy can also be used for work, like during muscle contraction 31. However, and this is important here, at least a portion of metabolic energy released during enzymatic cleavage of the phosphoanhydride bond is reused as biologically useful and applicable metabolic energy 10. Therefore, it was obvious to determine whether during the enzymatic cleavage of the phosphoanhydride bonds in polyP by ALP, part of the chemical energy released is utilized to form high-energy linkages in other compounds. In fact, experimentally it was shown that, after addition of polyP to cells cultured in medium/serum, ADP and also ATP are formed. Based on inhibitor studies it was elucidated that first ADP is produced, followed by an up-phosphorylation of ADP to ATP via the adenylate kinase (ADK) 32. The background concentration for these nucleotides in medium/serum is only ≈1 pmol/mL 33. This amount is insignificant compared to the level of ATP (650 pmol/mL) and ADP (20 pmol/mL) released in the extracellular medium/serum of SaOS-2 cell cultures (10^6^ cells/mL) after incubation with polyP 34, 35. This qualified polyP an extracellular generator of ATP using the enzymes ALP and ADK as catalysts. A similar, but enzymatically different ATP generation process was described in bacteria, in Acinetobacter johnsonii 36. There, the bacterial polyphosphate:AMP phosphotransferase, an enzyme that does not exist in eukaryotic cells, produces ADP from where ATP is formed via ADK.

In conclusion, using the ATP metabolism in muscles as a paragon for the metabolic conversion of ATP to the different energy forms 37, the chemical energy stored in the energy-rich bonds of polyP, like in ATP, and released from these molecules can be split into three parts of energy: metabolic (biochemically applicable) energy, heat (Q) and work (W) 38, 39. This triad can be expressed in terms of the change in enthalpy (ΔH), which is the sum of the change in internal energy (ΔU) and the product p•ΔV (enunciating the pressure multiplied by the change of the volume of the system), and as the change in Gibbs free energy (ΔG), by ΔG = ΔH-T•ΔS, where S stands for entropy. If pressure-volume work (p•ΔV) is excluded, which is the case for most chemical reactions proceeding in biological systems like tissues, ΔH equals ΔU. Since the change in enthalpy (ΔH) is partly due to the change in entropy (ΔH = ΔG + T•ΔS), which only contributes to the thermal change of the system, it is not possible to convert ΔH completely into other forms of energy than heat; consequently, only part of the change in Gibbs free energy (ΔH-T•ΔS) is available as metabolic energy or for conversion into work (mechanical, electrical, osmotic, etc.) and the residual sum is released as heat.

In addition to its function as a storage for metabolic energy, polyP has another functional property; it forms a coacervate in the presence of Ca^2+^ and Mg^2+^ 9, 40. These macromolecular assemblies, which form an aqueous, gelatinous, polymer-rich phase, can be prepared from either soluble Na-polyP or particulate Ca-polyP-NP 9 at pH 7. Coacervates are formed by phase separation from anionic polyP layers and cationic Ca^2+^ clusters and are never in a true thermodynamic equilibrium 41. Such processes are physiologically relevant because they are involved in dynamic intra- and extracellular compartmentalization sequences and, even though relevant to supramolecular polymer organizations, are also energy-dependent 42.

Although recognized as a driving force for the formation of membranes and artificial cells, the impact of coacervation on the function of cells has not been studied in detail. In this context, polyP is becoming increasingly important because the polymer has distinct morphogenetic effects during hard tissue formation such as bone and teeth 43, 44. Even more, coacervates have been shown to provide suitable delivery systems that can be applied, e.g., for delivery of bone morphogenetic protein 2 in bone regeneration 45. It is striking that the occurrence of liquid-liquid separation during coacervate formation is associated with strong movements of phases of different densities against each other. A study directed towards an understanding of the turbulent kinetic energy generated during this process has not yet been given. In model systems some progress has been reached 46. The energy expenditures, based on turbulence forces, during tissue formation, like during embryonic development or tumor metastasis, have been stressed but functionally not disclosed 47. In the present study it is outlined that coacervation has a distinct effect on the pattern formation of hydroxyapatite (HA) mineral on SaOS-2 cells.

Bone formation and bone growth are controlled by changing thermodynamic-driving forces especially at the growth regions 48. From a holistic perspective, bone formation is an extremely energy-demanding process that requires high levels of energy in the form of ATP, which is generated intracellularly during oxidative phosphorylation in the mitochondria of the osteoblasts 49. ATP is the decisive metabolite that controls cellular bioenergetics through interaction of ATP, ADP and/or adenosine with distinct purinergic receptors (reviewed in: 50). The production of heat in the muscles around the bone tissue in response to ATP hydrolysis is well established 51, and it can be postulated that the key metabolite ATP in bone, as a component of the musculoskeletal system, generates heat as well 52. HA is deposited in the extracellular space on the osteoblasts 53. Therefore, the pressing question arises as to where the extracellular ATP comes from. The physiological and pathophysiological growth of bone tissue proceeds in a close vicinity to vascularization a process in which blood platelets and megakaryocytes accumulate in the growth zones 54. In concert with this mineralization process the platelets are activated, which results in the release of polyP into the extracellular space. The polymer is needed for the stabilization of amorphous Ca-phosphate, which is the precursor of crystalline bone Ca-phosphate 55. A subsequent hydrolysis of polyP by ALP results in the transition of the amorphous to the crystalline phase of Ca-phosphate 44, 56. This mechanism would also explain the observation that ATP interferes with the growth of bone minerals on the osteoblasts 57; inhibition of ALP leads to freezing of the amorphous state of Ca-phosphate.

In the present study, the contributions of the three major biological energies, metabolic energy, mechanical energy and heat, to regenerative/reparative and vital processes occurring during wound healing and mineralization are examined. These processes have in common that they are driven by ATP, a metabolite produced after release of polyP in the extracellular space. In addition, when analyzing the polyP coacervation process, it became overt that, in addition to metabolic energy (ATP), the other two forms of energy, mechanical energy and heat, are likewise driving forces, especially during development and regeneration. In turn, the quantitation of the triad metabolic energy - mechanical energy - heat is proposed as a prognostic factor for favorable and individualized therapy of injuries in human medicine.

## Material and Methods

### Materials

Na-polyphosphate (Na-polyP) was prepared in our lab using Na-dihydrogenorthophosphate (NaH2PO4) as starting material as described 58. It is essential that in the thermal process used, a rapid cooling step is carried out at the end of the heating period in order to obtain an amorphous polyP product. Under these conditions, a polyP preparation is obtained which, after dissolution, does not shift the pH of the incubation medium. The material had an average chain length of about 30 phosphate (Pi) units.

The Ca-polyP-NP, which were determined to be amorphous by X-ray diffraction, were prepared as described 21 by mixing of Na-polyP with a two-fold higher molecular weight ratio of CaCl2•6H2O (Roth, Karlsruhe; Germany). The dried particles were termed Ca-polyP-NP. They had an average size of 100 nm.

All samples applied for human wounds were sterile filtered or treated with ethylene oxide gas.

The punch biopsy samples were extracted with Trizol (Fisher Scientific, Schwerte; Germany)/chloroform 59 and analyzed by 15% polyacrylamide gel electrophoresis (PAGE) containing 7 M urea 60. The gels were stained with toluidine blue O (#198161 Sigma) as outlined 61. A size marker of Na-polyP with a Pi chain length of 40 was run in parallel.

### Clinical issues

Ethics: To re-establish health after failure of standardized therapeutic attempts, unproven interventions in clinical practice with patients, following §37 of the Declaration of Helsinki, were performed 62. Written and signed informed consent was obtained for off-label use/application. The wound exudate was considered as scrap material and was granted by the ethics committee of Rhineland-Palatinate (21.09.2022).

Wound dressings: The hydrogel wound dressing was prepared from a suspension of hydroxyethyl cellulose (HEC; Caelo, Hilden; Germany; #4482 - NATROSOL250 HX Pharm) in 10% (final concentration) 1,2-propandiol (#2554; Caelo), which was supplemented with 65 mL purified water (#7732-18-5; Caelo) and 10 mL of phosphate buffer at pH 6.5 to a final concentration of 3%. The solution was then heated to 121°C for 20 min. Finally, 10 mL of the polyP sample were added and blended by rolling for 45 min, resulting in a final concentration of 600 μg/mL of Na-polyP and 60 μg/mL of Ca-polyP-NP. The final pH was adjusted to 6.5. The wound-hydrogel was stored in 10 mL syringes (# 4617100 V; B. Braun, Melsungen; Germany) with a Luer Lock adapter and sealing caps. Details have been given earlier 63.

For the collagen-based mats, collagen (type I) from cows (Lando Biomaterials, Shenzhen; China) was used. The material (10 mg collagen/mL) was dissolved in 20 mM acetic acid (pH 2.8) at 4°C for overnight 64. After neutralization, an 8 mL aliquot was poured into a 50 mm Petri dish and incubated at 37°C for 12 h. Then 64 mg solid Ca-polyP-NP were added together with gentamicin (2 µg/mL final). Finally, the mats were transferred into PBS (phosphate buffered saline) and incubated at 37°C for 12 h. The samples were then overlaid with ethanol (70% v/v) and pressed for 1 h until the formed 8 mm sized collagen mats had a thickness of 1 mm 64, 65. They contained ≈5 mg of Ca-polyP/g of wet mat.

The wetting solution was based on 120 mM phosphate buffer (pH of 6.5) and 20% (v/v final) propylene glycol (#398039, Sigma-Aldrich) as described 64. Finally, the polymer was added; Na-polyP (300 μg/g (w/w final) for immediate release of the biochemical activity) and 30 μg/g (w/w) Ca-polyP-NP as the depot form. Ten mL aliquots were filled (Sterifix 0.2 μm; B. Braun, Melsungen; Germany).

As an example of human chronic wound treatment with polyP, a patient was selected for whom both the polyP mat and the polyP hydrogel could be applied. The 78-year-old female suffered from a widespread, ulcerative, diffuse and deeply infiltrating basal cell carcinoma for over 6 months. The tumor was located on the capillitium and comprised a thickness of 4.4 mm. Three excisions were needed. Since the wound extended to the os parietale, the application of a collagen mat containing Ca-polyP-NP was indicated. In addition, the bandage was soaked with polyP wetting solution in order to activate tissue granulation. In the following, bandage change with ointment gauze was carried out every 3 d, followed by polyP hydrogel treatment. The final therapy was a split-thickness skin graft, which led to complete epithelialization.

The wound dressings applied were colonized with cells. For their investigation, collagen mats from patients, enriched with Ca-polyP-NP, were transferred to McCoy's medium /FCS and incubated there for 12 h. The cells growing out from the mats were further incubated in a polyP-free culture system or transferred into medium/serum supplemented with polyP, as indicated in the experiments. Finally, the cells were inspected by Nomarski interference contrast microscopy.

The wound fluid was collected by careful removal of the collagen mats from the wounds, 3 d after the last application of wetting solution. Subsequently, the specimens were used for the determination of either (i) ATP in the extracellular fluid or (ii) coacervate formation. During the incubation period, the samples were incubated in McCoy's medium/FCS comprising, if indicated additionally 5 mM Ca^2+^. The cells present in the mats were fluorescently labeled with Calcein-AM (#C1359, Sigma) and then optically counted in parallel using the Neubauer counting chamber (Roth, Karlsruhe; Germany).

To determine the morphology of cells in horizontal sections through mats and granulation tissue, punch biopsies were taken from collagen mats covering chronic wounds around three weeks after the start of wound treatment, with the aim of identifying the morphologies of fibroblasts and/or myofibroblasts. Those mats that had already been grown into the regenerating granulation tissue were selected. To distinguish these two cell types, cells were stained with rhodamine/phalloidin (reacts with the F-actin cellular cytoskeleton) and DRAQ5 (nuclei) as described 64, 66.

### Cell cultures and analytical studies

SaOS-2 osteoblast-like cells 67 derived from a human osteogenic sarcoma were cultured in McCoy's medium (containing 1 mM CaCl_2_) with 5% heat-inactivated fetal calf serum (FCS), 2 mM l-glutamine and gentamicin (50 µg/mL) in 6-well plates (surface area 9.5 cm^2^; Orange Scientifique, Braine-l'Alleud; Belgium). The seeding density was 5•10^3^ cells/cm^2^ in a humidified incubator at 37°C and 5% CO_2_ 68.

To activate the SaOS-2 cells for hydroxyapatite mineralization, the cultures were incubated in 24-well plates (Sigma) at a seeding cell concentration of 5×10^3^ cells/mL on glass discs for 2 d (cell concentration of 4×10^4^ cells/mL). The cultures were then incubated with 50 µg/mL of Na-polyP in McCoy's medium/FCS for the complete incubation period. To induce the cells to mineralize, the medium/serum was supplemented with the mineralization activation cocktail (MAC; 5 mM β-glycerophosphate, 50 mM ascorbic acid and 10 nM dexamethasone) 69. After additional incubation for 5 d, the cells were used for analysis.

To determine the effect of flow on mineralization, the SaOS-2 cells (1×10^6^ cells/mL) were embedded in the alginate matrix or seeded on top of this matrix as described 70. The matrix was composed of 4% (w/v) Na-alginate (W201502; Sigma) in 150 mM NaCl and was then mixed with McCoy's medium/FCS to reach a final concentration of 2% alginate. The matrix was hardened with 100 mM CaCl_2_ for 2 min. In two separate assays, the cells were either embedded in the matrix or placed on top of it. To start coacervation, Na-polyP, present either in the matrix (100 µg/mL) or in the culture medium/serum, was added together with 10 mM CaCl_2_.

The ATP pool was determined both in the wound exudate with the cells therein and in SaOS-2 cell cultures by quantifying the ATP-monitoring luminescence 35, 71. For the determination of ATP in wound exudate usually ≈100 µL of the fluid was added per well in 24 well plates. Then Na-polyP was supplemented reaching a final concentration of 10 µg/mL. Finally, ATP was determined with a luciferase-based assay after an incubation period of 30 min and 60 min respectively. The enzymatic ATP luminescence kit (no. LL-100-1; Kinshiro, Toyo; Japan) was used 72. The ATP level is given as pmol/mL. The wound exudate was collected from chronic wounds or from healthy individuals; per group n=6. For metabolic imaging, a described bioluminescence imaging technique 73, 74 was applied. SaOS-2 cells were preincubated for 4 d until they reached a density of ≈ 1•10^5^ cells/mL. The cells were then transferred to cover glasses and incubated in a basic solution of 60 g/L gelatin, 0.3 M glycerol and 30 g/liter polyvinylpyrrolidone (PVP) in a buffer solution (pH 7.6). To record ATP/ADP, the solution was supplemented with 44 U/mL hexokinase, 55 U/mL glucose-6-phosphate dehydrogenase, 13 mU/mL luciferase (from Photinus pyralis; # SRE0045, Sigma) and 8 U/mL NAD(P)H-FMN-oxidoreductase. Light reaction of bioluminescence for ATP was recorded with a highly sensitive computer-controlled single photon recorder (Argus 100 Photon Counting System; Hamamatsu, Herrsching, Germany) linked to a microscope (Zeiss) to record light emission. The different ATP levels were recorded semi-quantitatively (highest level: dark blue; lower ones: red/yellow).

Heat flow was measured at two different incubation temperatures; at 3.5°C and at 37°C (both at pH 7.0). Na-polyP (100 mg/mL) was added dropwise to a Ca^2+^ solution of 250 mg/10 mL over a period of 10 min with low-speed stirring. The temperature shifts were determined for 200 min and 90 min, resp., until a plateau was reached 75, 76. The temperature change was measured with a P750 temperature-humidity instrument (Dostmann Electronics, Wertheim; Germany - type P750; accuracy ± 0.03°C) linked to a PT100 sensor.

Microscopic analyses were performed with an environmental scanning electron microscope (ESEM) from Philips (Eindhoven; The Netherlands) or an SEM microscope (Zeiss Gemini; Oberkochen; Germany) 77. The double-detector system Auriga FE-SEM microscope (Carl Zeiss, Oberkochen; Germany) was used to determine the element distribution on living cells 78. Light microscopic images were recorded with a VHX-600 Digital Microscope (Keyence, Neu-Isenburg; Germany).

### Statistics

After calculating that the values are distributed according to standard normal Gaussian manner and the variances of the respective groups are equal, the results were statistically assessed using the independent two sample Student's *t*-test 79.

## Results and Discussion

### The basic theranostic biomarker for chronic wound healing: ATP-generating activity in wound exudate

**Figure 1:**
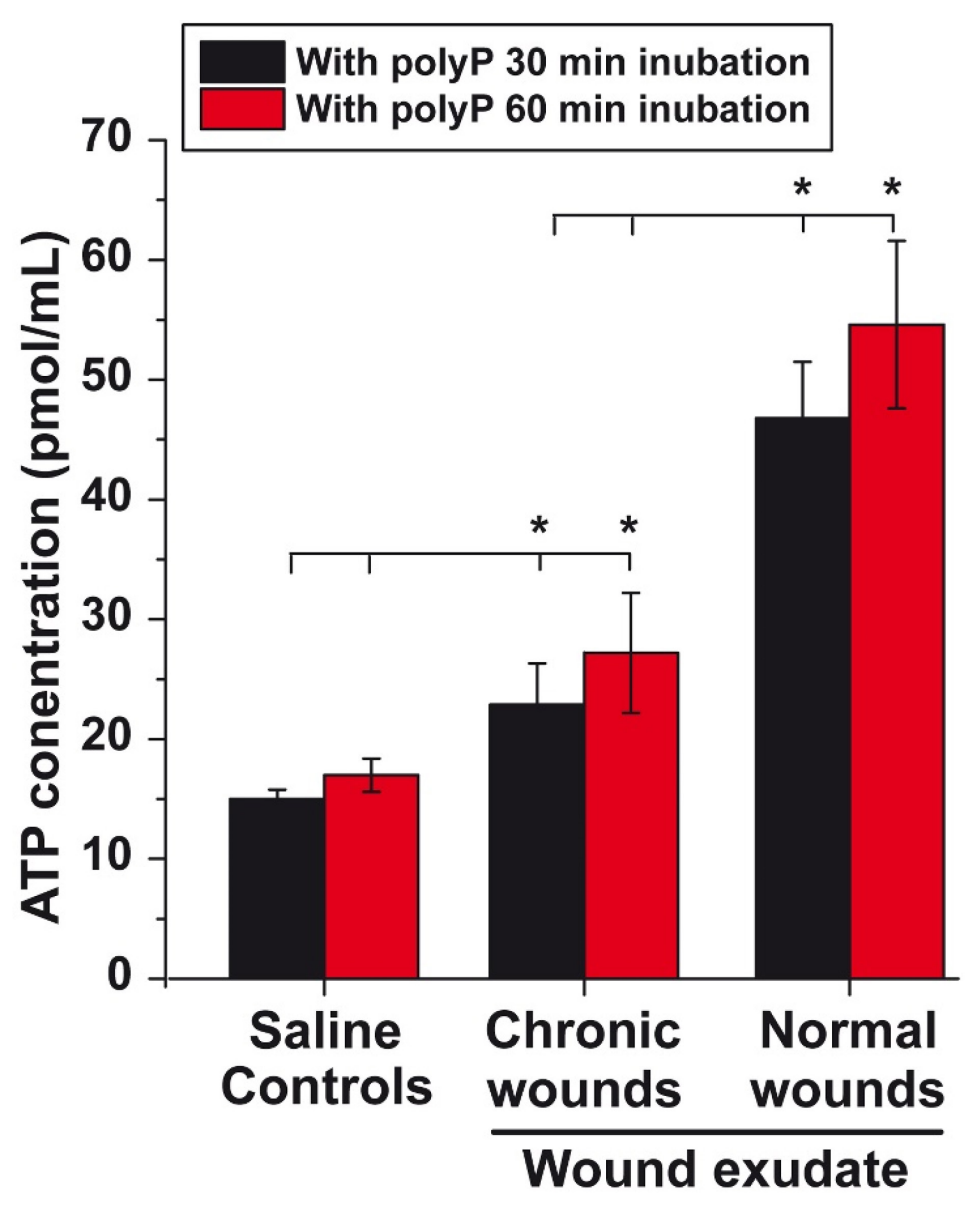
ATP level in wound exudate. As a control, an aliquot of wound exudate from healthy individuals was centrifuged prior to testing in the luminescence assay after supplementing with 10 µg/mL Na-polyP; the resulting sediment was suspended in saline and tested. Similarly, the aliquots of wound exudate from chronic wounds and from healthy individuals were tested (n=6). Means (in pmol/mL) ± SD are given; the significance is indicated (* P < 0.01).

In previous studies, we learned that the addition of polyP to wound dressings accelerates the healing of chronic wounds 63, 64. To overcome the challenges of poor wound diagnosis and limited clinical efficacy especially in chronic wound management, this contribution aims to highlight ATP generated from polyP as a theranostic biomarker for efficient therapy of chronic wounds. This aspect has not been sufficiently stressed before 80. It is the wound exudate that accomplishes important functions during the healing process, such as moistening the wound and supporting granulation and regeneration of the damaged tissue 81. The composition of the wound exudate is essentially similar to that of serum 82. In addition to cellular components such as leucocytes, platelets, macrophages, neutrophils and bacteria, the wound fluid contains cytokines and enzymes (lysozyme or matrix metalloproteinases) as well as ALP 83 and most likely also ADK 84. To assess the potential of the wound exudate to generate ATP, the wound fluid was assayed for ATP levels in the fluid phase using an ATP-monitoring luminescence assay 81. The data show that after centrifugation of the wound exudate (healthy individuals) and subsequent suspending of the sediment in saline, the background after a 30 min or 60 min incubation period in the presence of 10 µg/mL Na-polyP shows an ATP level of ≈15 pmol/mL, a concentration at which polyP causes a significant increase in cell growth 85. The ATP level in the exudate from chronic wounds is low and close to the control (Figure 1). Within this extracellular fluid, the cells and enzymes present in the medium produce an extracellular ATP level of 23 pmol/mL (30 min), which is significantly higher than the level in the saline medium. This value does not increase significantly when the incubation period is extended from 30 to 60 min in the presence of Na-polyP (Figure 1). However, when the exudate of normal wounds from healthy patients is tested under otherwise identical conditions, the ATP level increases 2.5-fold to ≈50 pmol/mL. Interestingly, the level measured in the fluid from chronic wound is within the range measured in the mesenchymal extracellular space 86.

### Healing success after application of polyP in collagen-wound coverage: The importance of the polyP coacervation

Two forms of application of polyP to chronic wounds were chosen in dependence on the depth of the wound. For deep wounds that reach down to the bone, a collagen-matrix wound coverage was selected. For more superficial injuries, a hydrogel-based treatment was fabricated. In both applications, polyP showed distinguished healing efficiency 63, 64.

**Figure 2:**
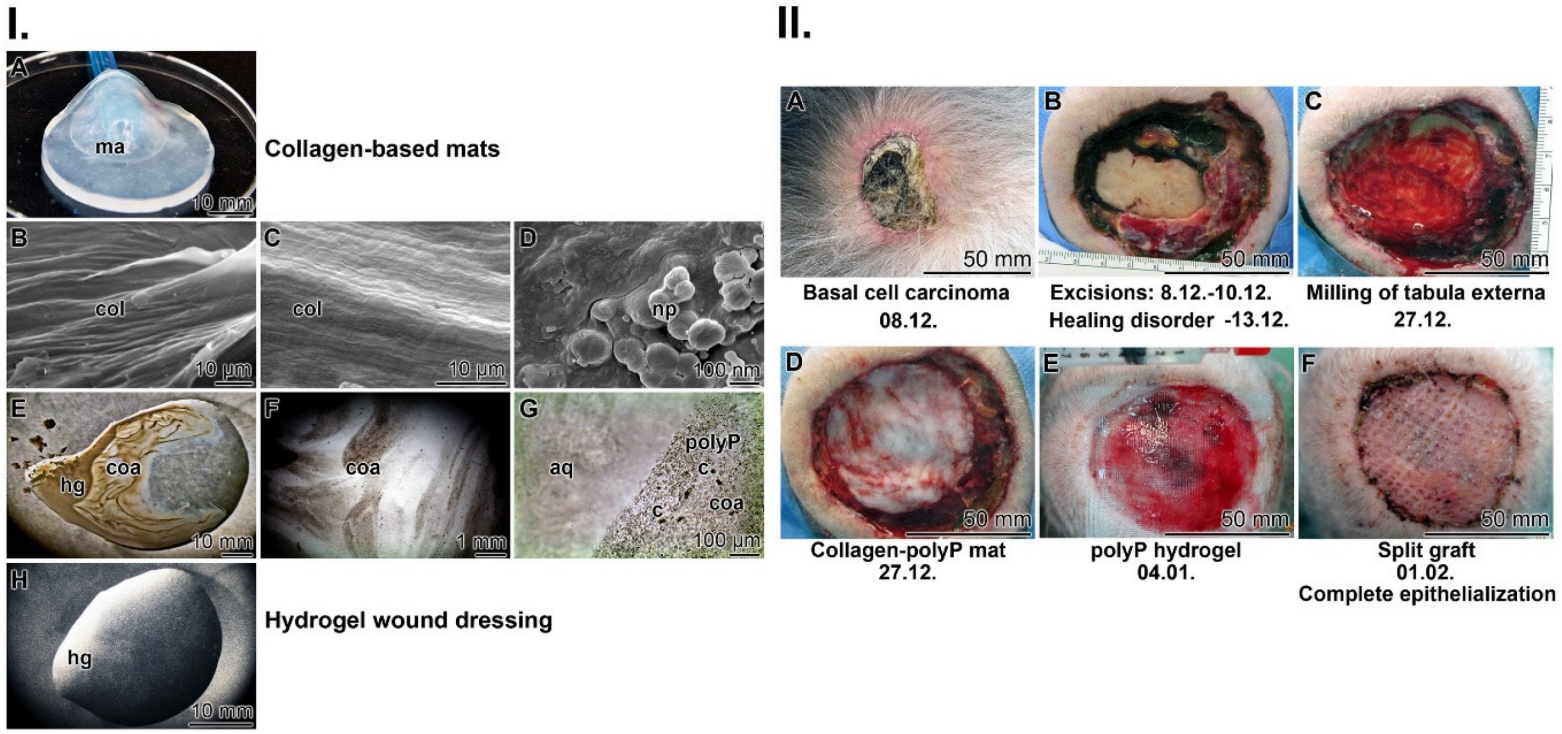
Fabrication of the two wound closures applied to human chronic wounds: (**I.**) (**A**) Collagen-based mat. Collagen, was pretreated by a pH shift (pH 3 to pH 7) to allow (**B** and **C**) a lateral alignment of the collagen bundles. The 1 mm thick mats (shown in A) were supplemented with polyP (5 mg of Ca-polyP-NP per g of wet collagen mat)*.* (***D***) The integrated NP (np) measured ≈100 nm. Separately, a wound gel (**E** to **H**) based on hydroxyethyl cellulose hydrogel (hg) was prepared with 600 μg/mL of Na-polyP and 60 μg/mL of Ca-polyP-NP. After transfer of the hydrogel into the wound exudate, the coacervate (coa) formation started, *(**G**)* a process during which the cells (c) within the exudate assemble in the polyP-rich (polyP) layers, which are separated from the aqueous layer (aq). (**II.**) Application of the two polyP wound coverages for the healing of chronic wounds in a human patient. (**A**) The initial deeply infiltrating basal-cell carcinoma tumor was extensively excised (**B**). Since the ≈80 mm large wound (**C**) did not heal (the cranial bone was still open), (**D**) a polyP-enriched collagen mat layered into the wound and every three days the lesion was moistened with the polyP-containing wetting solution. Now, rapid healing occurred and granulation tissue developed. Then, (**E**) treatment with the polyP-hydrogel started. The healing progress continued and (**F**) allowed a covering with a split skin graft. After 6 weeks of polyP treatment the patient was released home.

Collagen-based mats: Stable mats were prepared from cow collagen after a pH transition treatment (pH 3 to pH 7). The resulting collagen samples were supplemented with Ca-polyP-NP, transferred to PBS and then pressed into 1 mm thick sheets (Figure 2(I-A)). During this process, the collagen fibers within the mat align perfectly, become stable and surround the Ca-polyP-NP (Figure 2(I-B-D)). The size of the integrated NP remains at 100 nm during this course. The integrated polymer has previously been found to promote healing of chronic wounds.

Hydrogel wound dressing: The use of hydrogel-based wound dressings allows the absorption of wound exudate, protects chronic wounds against dehydration, accelerates degradation and, if included, delivers bioactive substances that promote regeneration (reviewed in: 87). As basis, the gelling and thickening properties of hydroxyethyl cellulose were exploited together with the active polyP polymer (600 μg/mL of Na-polyP and 60 μg/mL of Ca-polyP-NP); Figure 2(I-E-H). The inclusion of polyP in this hydrogel has been shown to enable fast healing of chronic wounds in an unprecedented way 63. It is especially Na-polyP in the hydrogel (Figure 2(I-E-H) that readily transforms into the active coacervate phase when the polymer comes into contact with wound exudate (Figure 2(I-F and G)). During this process, vortices appeared that separate the aqueous from the cell-rich flow zones ((Figure 2(I-G)).

The contribution of coacervation: Coacervation processes take place in an aqueous phase rich in macromolecules, with the molecules driven by ionic forces and concentration/diffusion gradients (Figure 2(I-F and G)). In the latter transitions, the conserved energy stored as potential energy is converted into kinetic energy. During this process, energy is dissipated, which can be sensed by G protein-coupled receptors, such as receptors for PTH and PTHrP, which control bone growth 88, 89. This example shows that turbulence forces acting during coacervation can be exploited for anabolic processes.

### Proof-of-concept: chronic wound healing

Here, to complement and support the remarkable successes in chronic wound healing reported in earlier studies by our group 63, 64, we describe another case reflecting the potential of polyP in wound healing. As described under Materials and Methods, a 78-year-old patient, who developed a deeply infiltrating basal cell carcinoma (Figure 2(II-A)), underwent three surgical resections of the tumor (Figure 2(II-B)). A tumor-free (T0) status was reached, which was associated with a bare calvaria. Impaired healing occurred that required further surgical removal of the partial tabula externa with a banded milling cutter, which simultaneously allowed debridement of the wound and induction of desired bleeding (Figure 2(II-C)). The wound was covered with a polyP-collagen mat (Figure 2(II-D)), which was additionally moistened with a wetting solution enriched with Na-polyP (for immediate release of polyP) and Ca-polyP-NP (as polyP depot). The wetting solution was renewed every third day over the complete regeneration period. Since partial regeneration occurred, polyP treatment was continued with a polyP hydrogel (Figure 2(II-E)). In response to this treatment, extensive granulation occurred, allowing the use of a split skin graft with a mesh size ratio of 1:1.5 (Figure 2(II-F)). Final healing was achieved by conventional treatment and the patient was able to leave the hospital.

This clinical proof-of-concept application clearly highlights the regenerative activity of polyP added to wound coverages, both together with collagen and embedded in a hydrogel. The causal metabolic energy delivering systems are addressed below.

In this context, it should be mentioned that in a previous wound healing study using mice, including diabetic animals, we also demonstrated that after addition of polyP to wounds, a significant upregulation of collagen gene expression occurs 90, which also reflects the presence of collagen fibers in the adjacent region around the wound. The latter result was obtained in animal studies using the calvarial bone defect model in rabbits, where histological sections were stained with Masson-Goldner to a light green area in the collagen/connective tissue 91. Using this model, it was also possible to show that wound regeneration started from the fringe of the wound by the formation of granulation tissue.

### Change in morphology of cells after transfer to polyP

The morphology of cells reflects their activity, function and mechanical properties 92. Less active cells have a roundish to elongated morphology, in contrast to actively regenerating cells, which are elongated and have processes projecting from their cell surfaces 93. Therefore, we examined the morphology of the cells associated with the wound bed and that of the cells present in the regenerative tissue.

**Figure 3:**
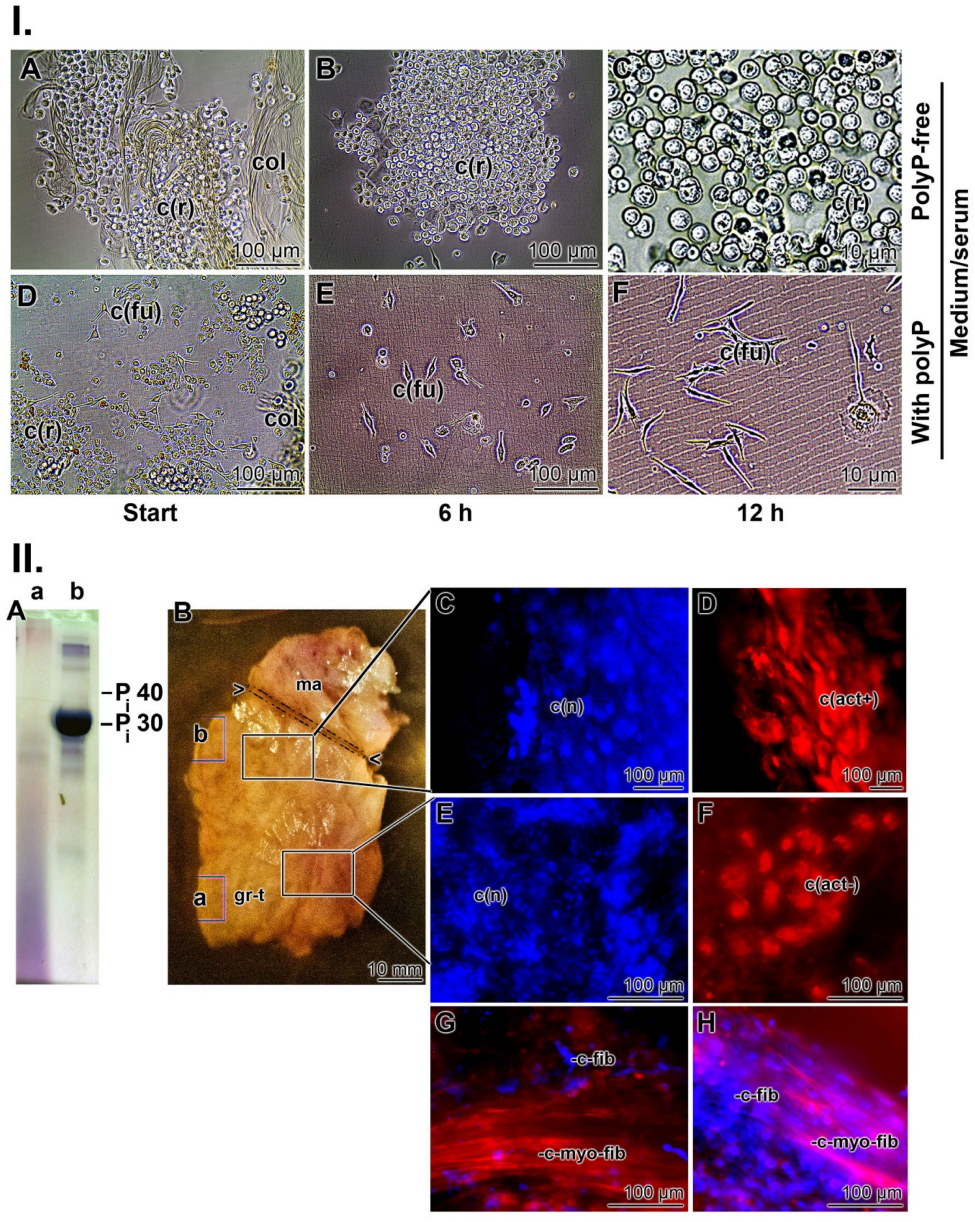
Characterization of the cells within the collagen-based mats and biopsy samples. (**I.**) Cells from collagen (col) mats used for chronic wound patients. (**A** to **C**) In polyP-free medium/FCS, the cells remain rounded (c(r)). (**D** to **F**) In contrast, after transfer of the cells into polyP medium/FCS (50 µg/mL Na-polyP), their morphology changed to an elongated/fusiform (c(fu)) shape during the 12 h incubation period. (**II.**) Biopsies taken from the collagen-based mats. The biopsies were dissected into two regions, a region adjacent to the polyP-enriched mat and the distal part embedded in the granulation tissue. (**A**) Using urea polyacrylamide gel electrophoresis (PAGE), (**A-a**) only little polyP is present in the distal region, whereas (**A-b**) a strong and distinct signal for polyP with a size of ≈30 P_i_ units is found in the section neighboring the polyP-mat. (**B**) Tissue section through the biopsy with the terminal mat (ma) and the distal regenerating granulation tissue (gr-t). The regions from where the samples were taken for PAGE are marked with (a) and (b), corresponding to the PAGE lanes in (A). (**C** and **D**) Histology after staining with DRAQ5-positive nuclei in blue (c(n)) and strongly actin-positive cells (in red); these spindle-shaped cells (c(act+)) are fusiform myofibroblasts. (**E** and **F**) In contrast, the fibroblasts within the regenerating regions have a rounded shape and lack distinct actin protrusions (c(act-)). (**G** and **H**) Longitudinal section through the distal regenerating granulation tissue area, highlighting the size differences between the fibroblasts (c-fib), measuring 10-15 µm, and the larger myofibroblasts with >60 µm (c-myo-fib) that were specifically stained for actin with rhodamine/phalloidin.

In the first series of experiments, collagen mats used to cover chronic wounds were transferred to medium/FCS and the outgrowing cells were inspected microscopically ((Figure 3 I)). In the absence of polyP, the spherical cells present in the collagen mesh maintained their morphology (Figure 3(I-A to C)), while in the presence of Na-polyP (50 µg/mL) in medium/serum, the cells changed to an elongated/fusiform shape (Figure 3(I-D to F)). This alteration reflects a high metabolic activity 94 and suggests the presence of myofibroblasts 95*.* The contraction and migration of myofibroblasts requires a considerable amount of ATP originating from cell metabolism 96. These strong energy-consuming processes depend on a physiological run of the citric acid cycle, coupled to the respiratory chain in the mitochondria, the intracellular site where metabolic energy (ATP) is generated.

Approximately three weeks after the start of wound treatment, punch biopsies were taken from collagen mats covering chronic wounds (mats together with regenerating tissue) and studied at the cellular and tissue level. A sign of intensive regeneration in wounds is the transition from fibroblasts to myofibroblasts 97. In contrast to fibroblasts with their smooth and regular surfaces and large nuclei, myofibroblasts are much larger (80 µm) and have undulating membranes and multiple processes 95. Therefore, the cells in the biopsies were stained with rhodamine/phalloidin (reacts with the F-actin cellular cytoskeleton) and with DRAQ5 (nuclei) as described 64, 66. The analyses were performed in two areas. First, the area next to the polyP mat and second, the section of the biopsy that was embedded in the granulation tissue. First, the polyP content was assessed by urea-PAGE electrophoresis. Using this approach, the strongest signal was seen in the section adjacent to the mat (Figure 3(II-A-b)), while the contents further distally only gave a smear (Figure 3(II-A-a)). The cells located in the upper region showed a typical myofibroblastic morphology. They are large spindle-shaped cells with DRAQ5-positive nuclei that were strongly stained when examined for the presence of actin fibers (Figure 3(II-C and D)). In contrast, the cells in the lower region, around the granulation tissue, have a rounded shape and their cytoskeletons are likewise rounded and do not have distinct protrusions (Figure 3(II-E and F)); they are most likely fibroblasts‑like cells that lack actin-positive filamentous structures 98.

Resting fibroblasts produce the extracellular matrix (ECM) in which these cells are in an inactive state 99. In response to activating stimuli, the fibroblasts become highly migratory, proliferative, and efficiently contribute to the regeneration and repair of damaged tissue 100. They enter the proto-myofibroblast stage, which is characterized by a rearrangement of the actin cytoskeleton from largely membrane-associated, monomeric G-actin into polymerized cytoplasmic F-actin stress filaments, the hallmark feature of the myofibroblasts 101. These cells are rich in non-myosin ATPase and therefore rely on the supply of metabolic energy. ATP and GTP are used for mechano-signaling via purinergic receptors and chemotaxis 102, 103. A characteristic distinguishing feature between fibroblasts and myofibroblasts is their cell size. While fibroblasts measure only 10-15 µm, myofibroblasts reach a length of over 60 µm due to their elongated processes, which are built of highly contractile α-smooth muscle actin 95, 104. These actin stress fibers are specifically stained with rhodamine/phalloidin; representative images are shown in Figure 3(II-G and H)*.*

In the ECM, polyP has been found to be the store for the production of ATP as the source of metabolic energy 39, which feeds the contractile apparatus and initiates the initial proliferative burst 105. Therefore, in the following sections, we addressed the question of whether the metabolic energy emerging from polyP/ATP only supports cell metabolism during wound healing or also includes other forms of energy such as energy for mechano-regulation during tissue remodeling or heat production 106.

The data presented show that the metabolic energy generated from polyP during enzymatic processes transforms fibroblasts into myofibroblasts, thus providing the platform for the migration of stem cells and endothelial cells into the wound 107. This property of directing cells toward the injury is crucial for regeneratively active cells. Furthermore, polyP and the downstream produced ATP upregulate wound cells to promote angiogenesis, as previously shown 32, 103. The use of polyP as an energy source is advantageous in particular for chronic wound healing, as ATP can be continuously synthesized from this polymer during bleeding, initially from soluble polyP (Na-polyP) and then from Ca-polyP-NP, which acts as a depot form of the polymer. This is particularly important because the ATP levels in the extracellular space are low and used up quickly 108.

### PolyP, the energy storing and dissipating polymer

Metabolic energy, in the form of ATP, is a limiting component in regeneration in humans 39, 109. The level of this metabolite is very high at 400 mM in the blood platelets, in which also polyP is stored 17. Surprisingly, the ATP level in the mitochondria is only ≈1 mM (reviewed in: 39). In the platelets, the levels of ADP and pyrophosphate are likewise high at 600 mM and 300 mM, resp. As mentioned above, the differences between the intracellular ATP level (around 5 mM) and the level of ATP in the extracellular space at only ≈20 nM are extreme 86. These figures underpin the notion that ATP in the extracellular space is a crucial, because limiting, factor, especially in regeneration processes. Furthermore, this finding suggests that, especially during regeneration, replenishment of ATP is required by administration of polyP, a polymer from which this metabolite is enzymatically formed 39.

ATP is not only the substrate for enzymatic phosphorylations or an overall anabolic component of the intermediary metabolism, but is also required for the physiological production of mechanical energy and the generation of heat 37*.*

**Figure 4:**
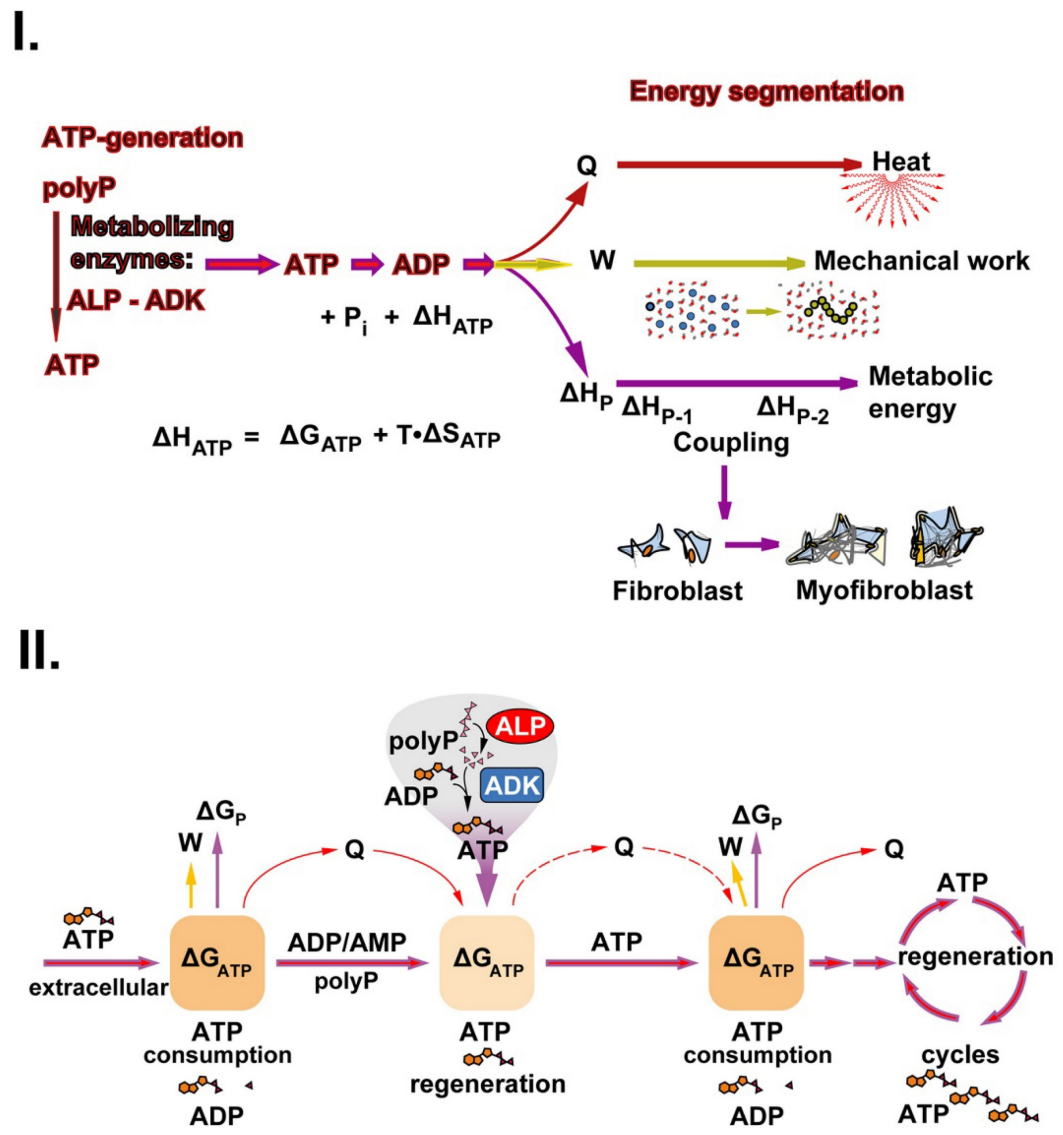
The energetic triad. (**I.**) ATP generates from polyP through enzymatic processing of polyP by ALP and ADK. The energy, stored in the phosphoanhydride bond of ATP (ΔH_ATP_) is split into energy used for heat production (Q), energy used for mechanical work (e.g., for cell migration) (W) and energy used for metabolic reactions (ΔH_P_). These three forms of energy drive the three energy segments heat production, mechanical work, and biochemical bond formation. (**II.**) ATP the key driver for chronic wound healing. Initially, the available low extracellular ATP pool (Gibbs free energy, ΔG_ATP_) is fueled into the energetic triad (heat energy, Q; energy for work, W; and metabolic energy used for metabolic reactions/product formation, ΔG_P_). When the extracellular ATP pool depletes, the polyP metabolizing enzymes ALP and ADK refill the depleting ATP pool. The produced heat (Q) is used for accelerating the biochemical reactions according to the Van 't Hoff equation and keeps the thermodynamic system running.

This triad is sketched in Figure 4(I). During the enzymatic processing of polyP, ATP is generated by ALP and ADK. When ATP undergoes dephosphorylation to ADP, this reaction is accompanied by an enthalpy change ΔH_ATP_. The energy gathered can be split into three fractions, energy used for heat (Q) production, energy for mechanical work (W) and finally, energy for metabolic reactions (metabolic energy), which usually occur in a coupled manner (Figure 4(I)). The fraction of ΔH_ATP_ converted into heat (Q) is equal to the sum of the entropic component (T•ΔS_ATP_) and the fraction of ΔG_ATP_ not converted into work (ΔG_ATP_ - W). The fraction of ΔH_ATP_ converted into work (W; excluding pressure-volume work) is equal to the fraction of ΔG_ATP_ not converted into heat, while the third fraction (termed ΔH_P_) is used for and maintained in new chemical bond formation in metabolic products (e.g., phosphotransfer reactions leading to phosphoester linkages). Heat energy released into the environment is used for thermal equilibrium of the biological system and mechanical energy work is utilized and applied, e.g., during the organization/arrangement of solid-state materials in biomineralization processes; usually those anabolic processes also require metabolic energy, e.g., for biosynthetic reactions. Exemplarily, an increased ATP production is crucial for the migration of myofibroblasts with their contractile apparatus.

Accordingly, for use in regenerative therapeutic treatments, polyP is required for patients e.g. during chronic wound healing to initiate and accelerate cell differentiation, adhesion, migration and subsequent granulation during regenerative tissue formation 110. In the initial phase of wound healing, the extracellular ATP is used as a source for energy, Gibbs free energy (ΔG_ATP_), which comprises, besides the entropy term (T•ΔS), also ΔH_ATP_. ΔG_ATP_ is converted into heat (Q), mechanical work (W), and metabolic energy until the metabolite ATP is consumed. At this stage, polyP replenishes the ATP pool with the help of the enzymes ALP and ADK (Figure 4(II)). After metabolic supplementation of ATP from the polyP reserves, ATP generation and coupled reaction cycles continue until the polyP stores are depleted. A driving parameter is also heat energy, which facilitates the metabolic reactions by mastering the activation energy barriers. This energy triad has been studied separately in the following sections.

### PolyP coacervate is the intermediate to the functional polymer

**Figure 5:**
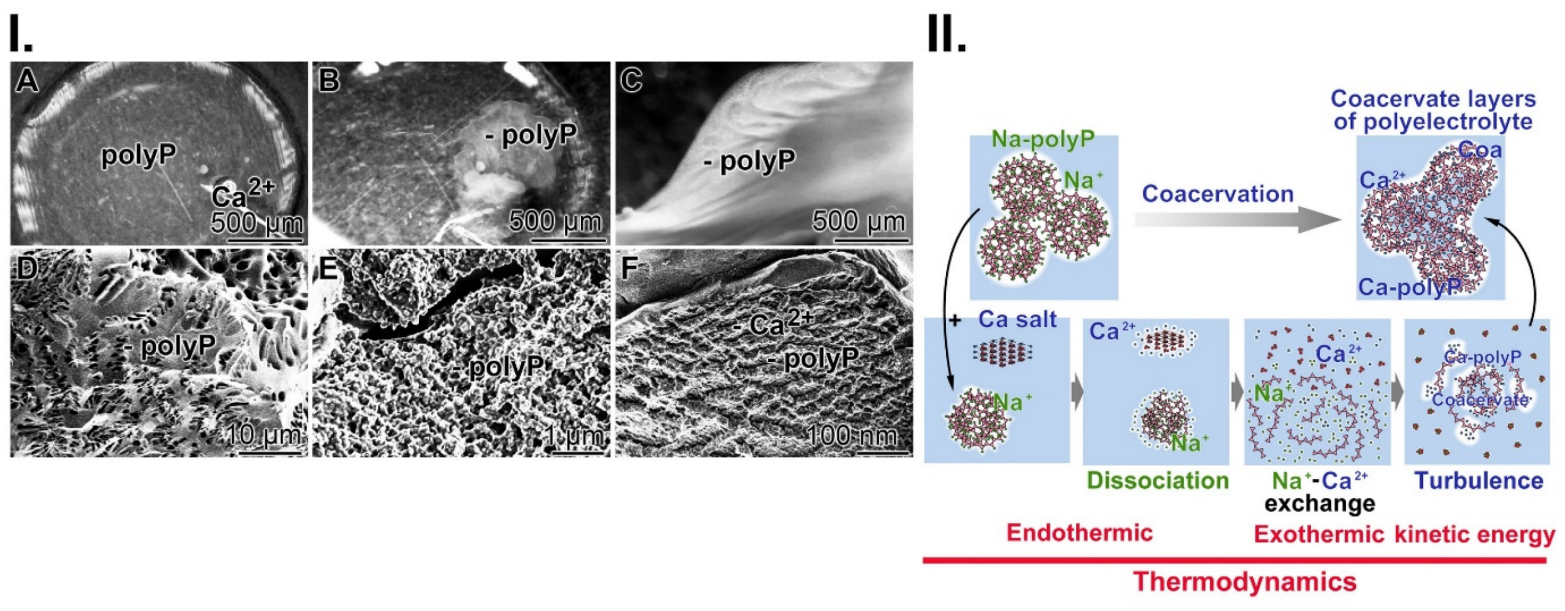
Coacervation, a dynamic process. (**I.**) Addition of Ca^2+^ ions to Na-polyP causes the formation (**A** to **C**) of the coacervate through a liquid-liquid phase separation, in which initially a denser phase composed of polyP and a more dilute phase (mainly Ca^2+^ ions) are formed; optical microscopy. (**D** to **F**) Finally more stable Ca^2+^-polyP ribbons are assembled; SEM. (**II.**) A schematic outline of the three phases during coacervation, dissociation (endothermic reaction), turbulence and final assembly in dynamic layers (exothermic reaction).

In previous studies, we have shown that polyP must be converted to the coacervate phase to become functionally active 9. Addition of Ca^2+^ to Na-polyP at pH 7 leads to the formation of a coacervate. During this transition, a liquid-liquid phase separation occurs, resulting in a polyP-rich denser phase and a more dilute phase of Ca^2+^ ions 40. This phase separation process is accompanied by intense turbulence motions, which are based on changes in the cohesive energy of the coacervate aggregates formed and lead to the observed rheological changes of the flow system (Figure 5(I)). The addition of Ca^2+^ to the Na-polyP solution results in immediate turbidity accompanied by the appearance of a turbulence current, which is due to the different densities of the phases formed by the opaque polyP flow and the intermittent transparent Ca^2+^ regions (Figure 5(I-A to C)). Turbulent kinetic energy is exploited in external or internal flow systems. There*,* turbulence plays a critical role in heat or mass transfer, dispersion or mixing. Turbulent kinetic energy supply provides energy to the turbulence, but a part of it is converted to kinetic energy keeping molecules in motion, during turbulent dissipation 111. In turn, turbulent kinetic energy supports the transporting properties of the myofibroblasts. Furthermore, this energy form adjusts the perfusion pressure and blood flow*.* Finally, this dynamic process of coacervate formation leads to a relatively stable separation into polyP ribbons and less dense Ca^2+^ zones (Figure 5(I-D to F)).

A reaction mechanism describing the process of coacervate formation is sketched in Figure 5(II). First, the dissociation of Na-polyP and CaCl_2_ into separate entities takes place. Secondly, the Na^+^/Ca^2+^ exchange occurs, followed by turbulence in the system and finally, coacervation, the separation of the reactants and products into two immiscible liquid phases, leading to a dense coacervate phase composed of aqueous Ca^2+^-polyP aggregates and a dilute phase containing the released Na^+^ and excess Ca^2+^ ions in equilibrium with the dense phase. The overall process is initially endothermic and then exothermic.

### Energy budgets during coacervation

**Figure 6:**
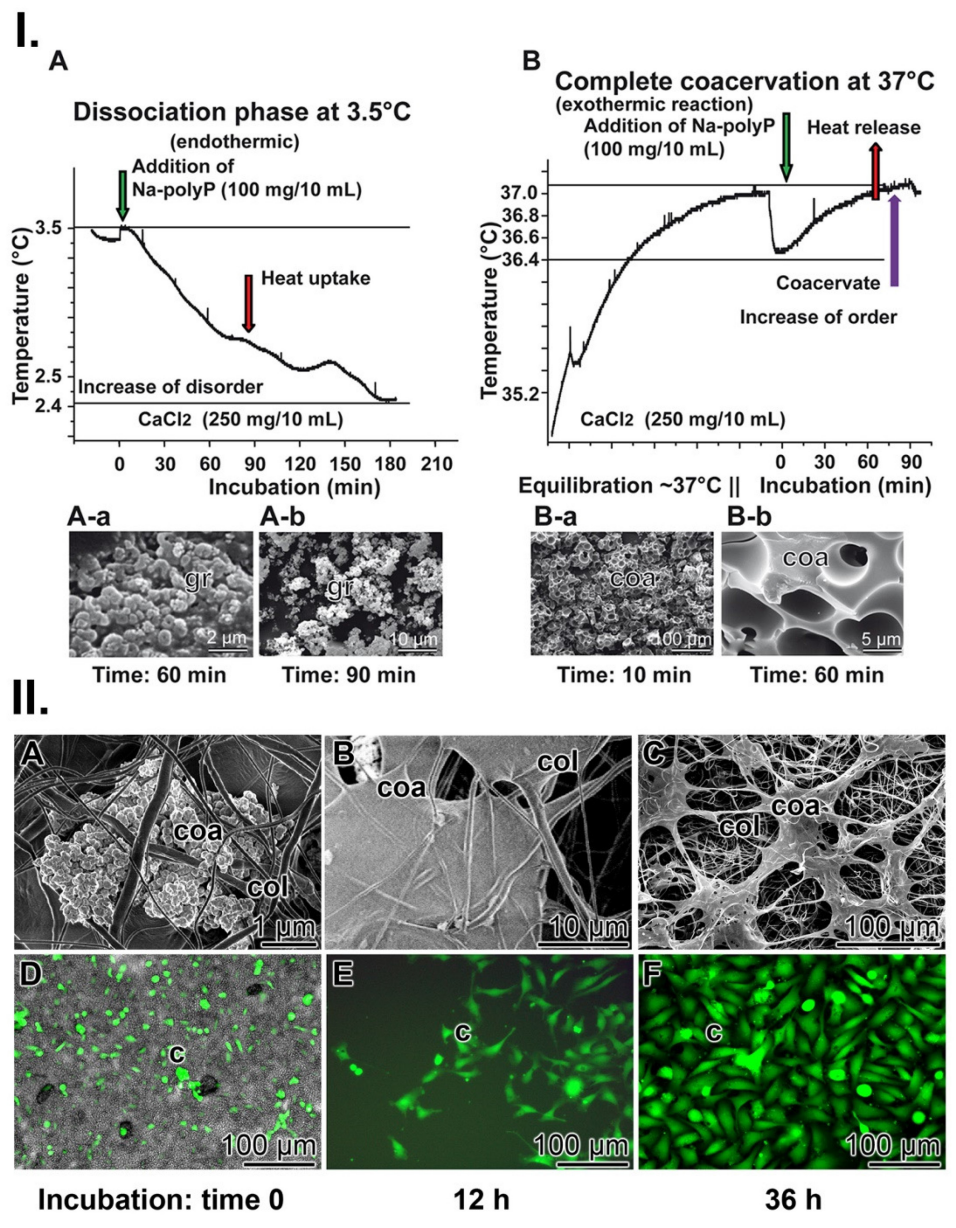
Coacervation process. (**I.**) Thermographic profile (**A**) at 3.5°C during the initial dissociation phase of the reaction, Na-polyP and CaCl_2_. During this endothermic phase, the temperature of the reaction mixture decreases (by 1.1°C). In this phase (**A-a** and **A-b**) no coacervates can be seen; only clusters of grains (gr) are visible. (**B**) The final phase of coacervation (at 37°C) is characterized by (**B-a** and **B-b**; optical microscopy) highly ordered coacervate blocks. This is an exothermic process and the temperature increases (by 0.7°C). (**II.**) PolyP coacervate formation in wound exudate. (**A** to **C**) PolyP-containing collagen mats are transferred to McCoy's medium/FCS and incubated for up to 36 h. During this period, coacervate (coa) clusters are rapidly formed; SEM. *(**D** to **F**)* Activation of cells in collagen mats from patients. Cells (c) residing in medium/FCS (with 50 µg/mL Na-polyP) change their morphology and turn from (D) spheroid to (E and F) elongated/fusiform, as seen after fluorescence staining with calcein AM.

PolyP coacervate formation can be dissected into two phases 112, 113. At first, the complex formation of oppositely charged ions/polymers by ionic bonds, and subsequently, the “aggregation” of the polymer layers to a coacervate; all in the aqueous medium. Coacervation is a temperature-dependent organized phase formation. In turn, to study the influence of temperature on coacervate formation, the reaction was performed both at a low temperature of 3.5°C and at 37°C (Figure 6(I)). Interestingly, during the reaction at 3.5°C, the temperature of the reaction mixture decreased from 3.5°C to 2.4°C over a period of about 3 h; ΔT 1.1±0.12°C (n=6). Consequently, at this temperature, the reaction is endothermic and the system absorbs heat energy, resulting in a positive ΔH (Figure 6(I-A)). Microscopic inspection revealed that under these temperature conditions no phase separation adjusts (Figure 6(I-A-a and A-b). Apparently, the reaction is delayed and not completed at this temperature. However, when the reaction was performed at 37°C, the temperature of the system did not decrease, but rather increased (Figure 6(I-B)). These results showed that at this temperature the progress of the reaction is exothermic (negative ΔH) and the formation of a coacervate was observed (Figure 6(I-B-a and B-b); ΔT 0.7±0.1°C (n=6). Surely, during this transformation, energy is released as heat but it is also implemented into the organization of the layers in the aqueous system.

It is assumed that during the dissociation phase at 3.5°C, the exchange of Na^+^ by Ca^2+^ ions at the polyanionic polyP takes place. This process involves the partial degradation of the hydrate shells of the Ca^2+^ ions and the formation of new hydrate shells around the liberated Na^+^ ions. Since Ca^2+^ ions bind the water molecules in their larger hydration shells more strongly than Na^+^ ions, more energy is required to overcome the solvation energy of the hydrated Ca^2+^ ions than for Na^+^ 114; the temperature of the system decreases. The Na^+^/Ca^2+^ exchange reaction is associated with an increase in entropy (positive ΔS), as the structuring effect on water molecules decreases due to the decreasing number of free (not bound to polyP) Ca^2+^ ions; the structuring effect of the Na^+^ ions released from polyP is smaller due their lower hydration number, compared Ca^2+^. Therefore, the first phase is driven by entropy (increase in disorder).

The second phase, leading to phase separation, is exothermic, most likely due to the release of energy (negative change in enthalpy) due to interactions within the coacervate structure formed (ionic interactions, H-bonding, Ca^2+^ complex formation between the polyP strands). Therefore, this process is enthalpically driven (negative ΔH) and accompanied by a negative entropy change (ΔG) due to the increased order caused by the coacervate formed.

### Coacervation of polyP incorporated into the collagen mats in the wound bed

To clarify whether polyP integrated into the wound mat undergoes coacervation during regeneration of chronic wounds, wound exudate was collected from the patient together with the polyP enriched collagen mats used as wound dressing. After transfer of the samples to McCoy's medium/FCS, containing ≈5 mM Ca^2+^, coacervate clusters formed (Figure 6(II-A to B)). During an incubation period of 12 h and 36 h, the gelatinous coacervate became more bulky. Even more, the cells present in these samples began to divide into large, continuous cell layers during this period (Figure 6(II-D to F)).

### PolyP as an efficient inducing polymer for hydroxyapatite patterning and ATP generation

**Figure 7:**
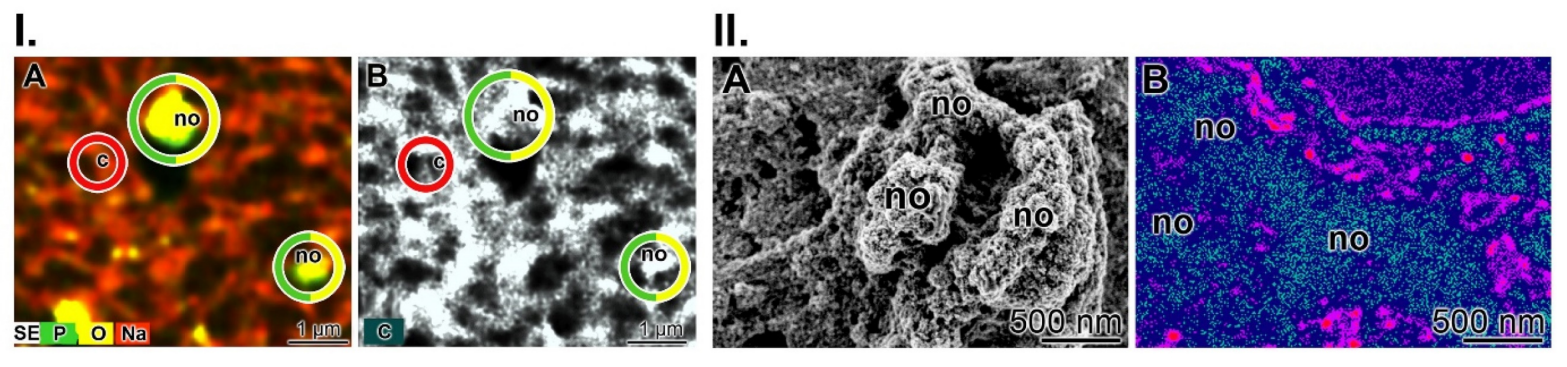
HA mineralization is paralleled with metabolic energy consumption. (**I.**) Element distribution on SaOS-2 cells during HA mineralization in culture medium. Two days after seeding, the culture medium was enriched with the mineralization activation cocktail MAC. (**A**) After incubation for 5 days, the cell layers were inspected using Auriga FE-SEM microscopy to assess the element distribution. The nodules (no) consist of phosphorus and oxygen, while the background with cells (c) is highlighted in reddish. (**B**) Using the secondary electron image technique, the nodules appear in grey. (**II.**) Regional ATP distribution across the surface of the *SaOS-2* cell layer. (**A**) Heavily mineralizing *SaOS-2* cells with their protruding mineral nodule (no) deposits. (**B**) Regional ATP distribution, assessed semi-quantitatively. The higher levels of ATP/ATP colored in dark/blue match with the nodule formations, while the surrounding regions are bright red.

As outlined above (Figure 3(I-D to F)) cells in the wound bed undergo, in the presence of polyP, a transformation of their morphology from the spherical phenotype to an elongated/fusiform appearance. An even more obvious change caused by polyP on the function and product formation can be objectivized with SaOS-2 cells during HA crystallite secretion in the presence of polyP 115-117. (Figure 7(I)). The mineral deposition process is induced by polyP after induction of tissue-nonspecific type alkaline phosphatase (TNAP) 68.

The element distribution of the mineral deposits on living cells was analyzed using the double-detector system Auriga FE-SEM microscope (Figure 7(I)). It showed strong signals for phosphorus and oxygen above the nodules (Figure 7(I-A)), while the background contained cells scanned for the sodium signal. In parallel, the secondary electron image of the analyzed area is shown; here the nodules do not flash in greyscale, but are blank (Figure 7(I-B)). In contrast, the cell areas are dark.

In the present study, we used the metabolic imaging technique to correlate ATP expenditure with the degree of mineralization (Figure 7(II)). The corresponding SEM image is shown in Figure 7(II-A) and the signals for imaging of bioluminescence for ATP are given in Figure 7(II-B). The ATP distribution along fully mineralized SaOS-2 cells was measured (pre-incubation for 4 d, followed by incubation for 5 d together with a mineralization activation cocktail (MAC)). According to this schedule, the cells exhibit extensive mineralization and have HA nodules on their surface. The size of the mineral deposits on each cell is ≈200 nm (Figure 7(II-A)). In parallel to this series, the cells were subjected to ATP metabolic imaging using bioluminescence cell microscopy (Figure 7(II-B)). It can be is seen that, in the pseudocolor image, the regions with intense mineralization, the nodule areas, are in dark/blue, indicating the highest level of ATP, while the surrounding regions are bright for red and cover low ATP regions.

### Effect of mechanical movement during coacervation on crystallite orientation

**Figure 8:**
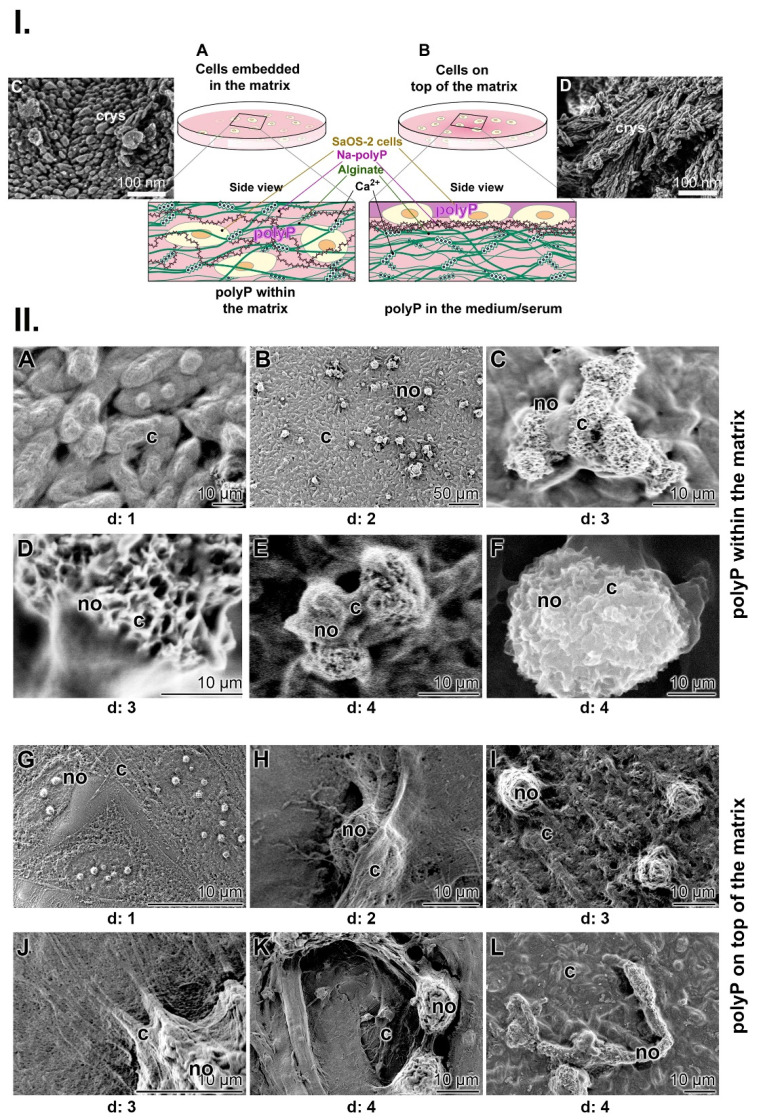
Influence of coacervation turbulence on the orientation pattern of the crystallites on SaOS-2 cells. (**I*****.***) Sketch of the experimental setup. (**A**) Cells were embedded in an alginate-based matrix, hardened with Ca^2+^ and enriched with Na-polyP (left panel); no contact of the cells is possible. (**B**) The matrix was prepared first without Na-polyP; then this polymer was added as an overlay to cells growing in medium/serum; coacervation was initiated by adding Ca^2+^. (**C**) Pattern of crystallites (crys) on SaOS-2 cells not exposed to coacervation. (**D**) Linearly arranged crystallites (crys) on the surfaces of cells within the coacervate vortices. (**II.**) Mineralizing nodules of cells within the alginate matrix and of cells on top of the matrix; SEM; ESEM. (**A** to **F**) The mineralizing nodules (no) of cells within the matrix, not exposed to the mechanical flow energy caused by coacervate formation, are arranged in a random pattern on the cell (c) layer (A to C) in the first phase at day 1 to day 3. As the mineral nodules grow, they fuse together (day 3 (d: 3) to day 4) and are (D to F) marginally separated by cell extensions. (**G** to **L**) The organizational pattern of the nodules is significantly different if the cells were directly exposed to the coacervate movements/fluxes. Initially, (G to I) the pattern of the nodules is again random (day 1 to day 3), while in the final phase (J to L) the cells change their morphology and the fusiform protrusions arrange the nodules in a threadlike pattern during the 4-day incubation period.

As mentioned, a physiologically relevant property of polyP is to undergo coacervation in the presence of divalent cations. During this process, the oppositely charged electrolytes become hydrated and separate from each other, existing hydrate shells are broken down and a Na^+^/Ca^2+^ exchange occurs, causing a dynamic and transient flow in the surrounding solution (Figure 5(I-C)). To visualize whether the mechanical forces thereby created influence the orientation of the ≈50 nm nanoapatite crystallites, which can also be observed during bone and teeth formation 118, the mineralizing SaOS-2 cells with the formed HA crystals were either directly exposed to the moving fluid during the coacervation process or the laminar flow was separated from the cells. To achieve this situation, the two processes were separated by an alginate barrier (Figure 8(I)). In turn, to suppress the mechanical shear force during the course of coacervation, Na-polyP (100 µg/mL) was either embedded together with the SaOS-2 cells in an alginate matrix in 24-well culture plates (Figure 8(I-A)) or the cells were layered together with the polymer on a polyP-free matrix (Figure 8(I-B)). The coacervation process was then started by addition of Na-polyP with 10 mM CaCl_2_. MAC was added together with CaCl_2_. SEM analysis showed that the crystallites formed by the cells directly embedded in the Na-polyP-supplemented alginate matrix remain in a disorganized pattern on the cell surfaces (Figure 8(I-C)). In contrast, when the cells were exposed to the coacervate on the surface of the alginate matrix (addition of Na-polyP and CaCl_2_ to the medium), the crystallites orient themselves in lines on the cells after addition of MAC (Figure 8(I-D)).

Even more, when polyP is enclosed in the alginate matrix, the mineralizing cells with their mineral nodules clump together. They aggregate into round-shaped groups during a 4-d incubation period (Figure 8(II-A to F)). The images also show the dynamics of the mineralization process, in which the cells encompass the mineralizing nodules with their extensions. In these mineralizing centers, the nodules can reach a size of up to 50 µm. On the other hand, when the mechanical forces are allowed to reach the cell surfaces during the coacervation, a completely different organization pattern of the nodules can be seen (Figure 8(II-G to L)). Initially, at day 1, the growing nodules are again randomly arranged on the surfaces of the cells (Figure 8(II-G)). However, in the following days (day 2 to day 3), the cells elongate and round-shaped nodules are formed, which are smaller in size of around 8 to 10 µm (Figure 8(II-I)). The cells not only elongate but also form filamentous protrusion (Figure 8(II-J)). Finally, the filaments with their fusiform protrusions drive the mineralization pattern toward a thread-like pattern during the 4-day incubation period (Figure 8(II-K and L)).

## Conclusion

Regenerative repair processes are complex with dynamic and high energy demands. Most often, it is the metabolic energy in the form of ATP that is spent on the formation of new covalent bonds. ATP thereby provides the energy for both energy-consuming endergonic reactions (non-spontaneous reactions; or reactions in coupled metabolic chains) and energy-releasing exergonic reactions (spontaneous reactions), which, however, also require an input of activation energy. When the high-energy bonds in ATP are broken, energy is released and can be harnessed for cellular work. During such metabolic conversions, two further forms of energy are or can be produced and released that are likewise important for regeneration processes: heat and mechanical energy. They are not waste forms of energy, but are equally important in transmitting extracellular signals to cells 119. The corresponding receptors, the thermoreceptors, mechanoreceptors and nociceptors, are inserted in the skin and at their nerve endings, which perceive warming and cooling stimuli, control epidermal homeostasis (proliferation of keratinocytes is modulated by skin innervation), and regeneration 119, 120.

While the effect of metabolic energy on tissue regeneration is well documented by the proof-of-concept healing success of chronic wounds in patients, the impact of mechanical energy and heat on the skin healing process is more difficult to demonstrate. In the present contribution, the HA mineralization process of SaOS-2 cells, which can be used as a template for understanding bone mineral formation, was used as an experimental model system to assess the role of the two forms of energy, mechanical energy and heat.

A distinguished property of polyP is its potential to form an aqueous coacervate phase in the presence of divalent cations 40. During this transformation process, fluctuations due to liquid-liquid phase separation occur, in which the dense and dilute phases, which are in thermodynamic equilibrium, move laterally along each other and create flow vortexes. Thermodynamically, the initial phase, the dissociation of the reaction partners to freely diffusible ions (polyP anions and Na+ cations) and the breakdown of the strongly bound and highly organized hydrate shells of the Ca2+ ions, requires energy, while the degree of disorder (entropy) increases. During the following ion exchange reaction (Na+/Ca2+), Ca2+ cations form ionic bonds with polyP and, in the final phase of the reorganization, cause the organization into the inverse laminar flow layers, exothermic reactions that are accompanied by an increase in order of the system (decrease of entropy) (Figure 6).

What was striking was the effect of the mechanical energy generated by polyP through the formation of the inverse laminar flow layers on the organization of the crystallites formed on mineralizing SaOS-2 cells. The vortexes generated during coacervation caused a laminar alignment of the crystallites along the stream layers formed by the coacervate (Figure 8 (I-C and D)).

Building on the energetic triad generated by polyP, metabolic energy, mechanical energy and heat, the successful proof-of-concept of polyP in healing chronic wounds (Figure 2(II)) can be explained. Metabolic energy is required for activation of fibroblasts to myofibroblasts, which provide a platform for the migration of wound cells during regeneration and granulation tissue formation. Heat in the extracellular medium contributes to overcoming the activation energy for enzyme reactions, thereby accelerating ATP generation from polyP. Mechanical energy is consumed for the formation of stronger and more organized scars 121 via components of the cell membrane and cytoskeleton, as well as signaling pathways that transmit mechanical signals to the cells and accelerate regeneration and the final stage of wound repair 122.

PolyP is an ancient polymer that most likely contributed to the building of the protocell, a self-organized, endogenously ordered precursor of cells in the origin of life 16. This polymer, in turn, has become a cornerstone for maintaining cell metabolism and is believed to have the potential to become a central molecule in medical regenerative tissue repair. Energy supply in the body via ATP generated in the mitochondria and also from the condensed energy store, polyP 39, with its three primary energy forms, metabolic energy, thermal energy and mechanical energy, drives intracellularly biochemical reactions. PolyP can also be considered as an extracellular hub for energy supply that, once released from platelets, provides energy for development and repair processes when needed 109.

In conclusion, the data presented here demonstrate that all three forms of energy, metabolic energy - mechanical energy - heat, are crucial for an efficient regeneration and reconstruction of damaged tissue. The task for the future is now to develop suitable, sensitive and reliable sensors to quantify the contribution of the individual components of this energy triad in order to enable the translation of these findings for the benefit of patients.

## Abbreviations

ADK: adenylate kinase
ALP: alkaline phosphatase
Ca-polyP-NP: Ca-polyP nanoparticles
FCS: fetal calf serum
HA: hydroxyapatite
HEC: hydroxyethyl cellulose
MAC: mineralization activation cocktail
NaH2PO4: Na-dihydrogenorthophosphate
Na-polyP: Na-polyphosphate
NP: nanoparticles
PBS: phosphate buffered saline
polyP: polyphosphate
SEM: scanning electron microscopy
W (work): mechanical energy
ΔG: Gibbs free energy
ΔH: enthalpy
ΔH: entropy
ΔU: internal energy
